# Exosomes released upon mitochondrial ASncmtRNA knockdown reduce tumorigenic properties of malignant breast cancer cells

**DOI:** 10.1038/s41598-019-57018-1

**Published:** 2020-01-15

**Authors:** Lorena Lobos-González, Rocío Bustos, América Campos, Valeria Silva, Verónica Silva, Emanuel Jeldes, Carlos Salomon, Manuel Varas-Godoy, Albano Cáceres-Verschae, Eduardo Duran, Tamara Vera, Fernando Ezquer, Marcelo Ezquer, Verónica A. Burzio, Jaime Villegas

**Affiliations:** 1 Centro de Medicina Regenerativa, Facultad de Medicina, Clínica Alemana-Universidad del Desarrollo, Santiago, Chile; 2 Andes Biotechnologies/Fundación Ciencia & Vida, Santiago, Chile; 3 Centro de Estudios Avanzados de Enfermedades Crónicas (ACCDIS), Santiago, Chile; 4 Facultad de Ciencias de la Vida, Universidad Andrés Bello, Santiago, Chile; 5 Laboratorio de Comunicación Celular, Programa de Biología Celular y Molecular, Instituto de Ciencias Biomédicas (ICBM), Facultad de Medicina, Universidad de Chile, Santiago, Chile; 6 Exosome Biology Laboratory, Centre for Clinical Diagnostics, University of Queensland Centre for Clinical Research, Royal Brisbane and Women’s Hospital, The University of Queensland, Brisbane, Australia; 7 Cancer Cell Biology Lab., Centro de Biología Celular y Biomedicina (CEBICEM), Facultad de Medicina y Ciencia, Universidad San Sebastián, Santiago, Chile; 8 Doctorado en Biomedicina, Centro de investigación e innovación Biomedica, Universidad de los Andes, Santiago, Chile; 9 Centro de Medicina Veterinaria, Facultad de Ciencias de la Vida, Universidad Andrés Bello, Santiago, Chile

**Keywords:** Cancer, Biomarkers

## Abstract

During intercellular communication, cells release extracellular vesicles such as exosomes, which contain proteins, ncRNAs and mRNAs that can influence proliferation and/or trigger apoptosis in recipient cells, and have been proposed to play an essential role in promoting invasion of tumor cells and in the preparation of metastatic niches. Our group proposed the antisense non-coding mitochondrial RNA (ASncmtRNA) as a new target for cancer therapy. ASncmtRNA knockdown using an antisense oligonucleotide (ASO-1537S) causes massive death of tumor cells but not normal cells and strongly reduces metastasis in mice. In this work, we report that exosomes derived from ASO-1537S-treated MDA-MB-231 breast cancer cells (Exo-1537S) inhibits tumorigenesis of recipient cells, in contrast to exosomes derived from control-ASO-treated cells (Exo-C) which, in contrast, enhance these properties. Furthermore, an *in vivo* murine peritoneal carcinomatosis model showed that Exo-1537S injection reduced tumorigenicity compared to controls. Proteomic analysis revealed the presence of Lactadherin and VE-Cadherin in exosomes derived from untreated cells (Exo-WT) and Exo-C but not in Exo-1537S, and the latter displayed enrichment of proteasomal subunits. These results suggest a role for these proteins in modulation of tumorigenic properties of exosome-recipient cells. Our results shed light on the mechanisms through which ASncmtRNA knockdown affects the preparation of breast cancer metastatic niches in a peritoneal carcinomatosis model.

## Introduction

Breast cancer is one of the most common cancers in women worldwide, as one in every eight females will be diagnosed with this disease in their lifetime^1^. Current treatments for this type of cancer are mastectomy, chemotherapy, radiation and hormone therapy, among others; but the downside of these treatments is that the rate of success is very low in advanced stages of the disease^2–4^, mainly because secondary niches in breast cancer are multi-tissue and, in consequence, the location of the residuary is unpredictable^5–7^. This second niche can localize to lymph nodes and distant organs such as ovaries, bone or spleen^5^. If it were possible to give a clear prognosis and predict the tisular location of new nodules, surveillance programs for this disease would be highly simplified and would largely benefit many patients.

Classic markers for breast cancer, such as Ki67, Estrogen Receptor (ER), Progesterone receptor (PR), Human epidermal growth factor receptor 2 (HER2), p53, ARF tumor suppressor (p14ARF), cyclin D1, cyclin E, T-box transcription factor 2 and 3 (TBX2/3), breast cancer type 1 and 2 (BRCA1/2), and vascular endothelial growth factor (VEGF)^7^ are no longer able to give a clear prognosis in the evolution of a patient’s disease and therefore other markers are currently being studied. New molecular targets such as CXCR4, CAV1, FOXP3 and microRNAs are promising candidates for the future development of effective and targeted therapies^7–9^, however, more studies are needed to use them in clinical practice, due to the heterogeneity of breast cancer metastasis^10,11^.

Communication between tumor cells plays a very important role in metastasis. Recent studies have shown that intercellular communication during this stage is mainly mediated by microvesicles, and specifically by exosomes^12–17^ which have been proposed to play an essential role in the preparation of metastatic niches and in the promotion of invasion^12–14^. Exosomes are membrane-derived extracellular vesicles (EVs), 40–150 nm in diameter, released by a variety of cell types. These EVs contain bioactive molecules, such as nucleic acids (DNA, mRNA, microRNA and other non-coding RNAs), proteins (receptors, transcription factors, enzymes, extracellular matrix proteins) and lipids, which can potentially redirect the function of a recipient cell^15–18^. Therefore, exosomes are emerging as local and systemic cell-to-cell mediators^17^ carrying oncogenic information that plays an important role in cancer progression^19–22^.

Exosomes secreted by metastatic cells potentiate tumorigenic capacities of less aggressive cells acting in a paracrine manner^23–27^. Moreover, exosomes are able to modify the microenvironment in recipient tissues of metastatic cells^20,21,28,29^, promoting angiogenesis in these secondary breast cancer niches^30^. Breast cancer-derived exosomes contain transforming growth factor β (TGF-β), which converts fibroblasts into myofibroblasts, contributing to vascularization, tumor growth and local invasion^31^. These exosomes also promote a myofibroblastic phenotype in adipose tissue-derived mesenchymal stem cells, resulting in increased expression of VEGF, stromal cell-derived factor 1 (SDF-1) and C-C motif chemokine ligand 5 (CCL5)^32^. Also, tumor-associated fibroblasts secrete exosomes which have been shown to promote tumor cell mobility, invasion and dissemination through the Wnt-planar cell polarity (PCP) pathway^33^. On these grounds, it is important to define the macromolecules (lipids, RNA or proteins) present in exosomes from tumor cells and their effect on the ability of recipient cells of conditioning the microenvironment to harbor metastatic cells.

Previous research by our group described a family of lncRNAs originated from the mitochondrial 16 S rRNA gene (ncmtRNAs). This family comprises sense (SncmtRNA) and antisense (ASncmtRNA-1 and -2) members, both with stem-loop structures^34^. While SncmtRNA is detected in all proliferating cells, ASncmtRNA is strongly downregulated in tumor cells from diverse origins, constituting a vulnerability of tumor cells, since transfection an antisense oligonucleotide (ASO) directed towards the ASncmtRNA (Andes-1537S) triggers massive (60-80%) apoptotic death in a wide variety of tumor cells, with no effect on viability of normal cells^34–37^. In addition, this approach significantly reduces tumorigenic and metastatic properties^34–38^, suggesting the antisense non-coding mitochondrial RNA (ASncmtRNA) as a new therapeutic target against tumor cells. In the MDA-MB-231 human breast cancer cell line, ASncmtRNA knockdown induces ~60% apoptosis at 48 h^38^. In this work, we show that, at 24 h, Andes-1537S-treated cells secrete exosomes (Exo-1537S), which trigger a reduction in migration, invasion and anchorage-independent growth capacity. In an *in vivo* mouse model of peritoneal carcinomatosis with MDA-MB-231 cells, treatment with Exo-1537S significantly decreased tumorigenesis, confirming our *in vitro* results. A differential proteomic analysis determined that S100A9, VE-Cadherin and Lactadherin were enriched in exosomes released from cells transfected with a control ASO (ASO-C) (Exo-C) and non-treated cells, but were undetectable in Exo-1537 S vesicles. The former, however, were enriched in proteasomal subunits. To our knowledge, this is the first report on the differential presence of these proteins in exosomes, which is interesting since these proteins are known to be involved in metastasis^39^ and could be involved in conditioning the metastatic niche.

## Results

### ASncmtRNA knockdown reduces viability and tumorigenic potential of MDA-MB-231 breast cancer cells

**Figure 1:**
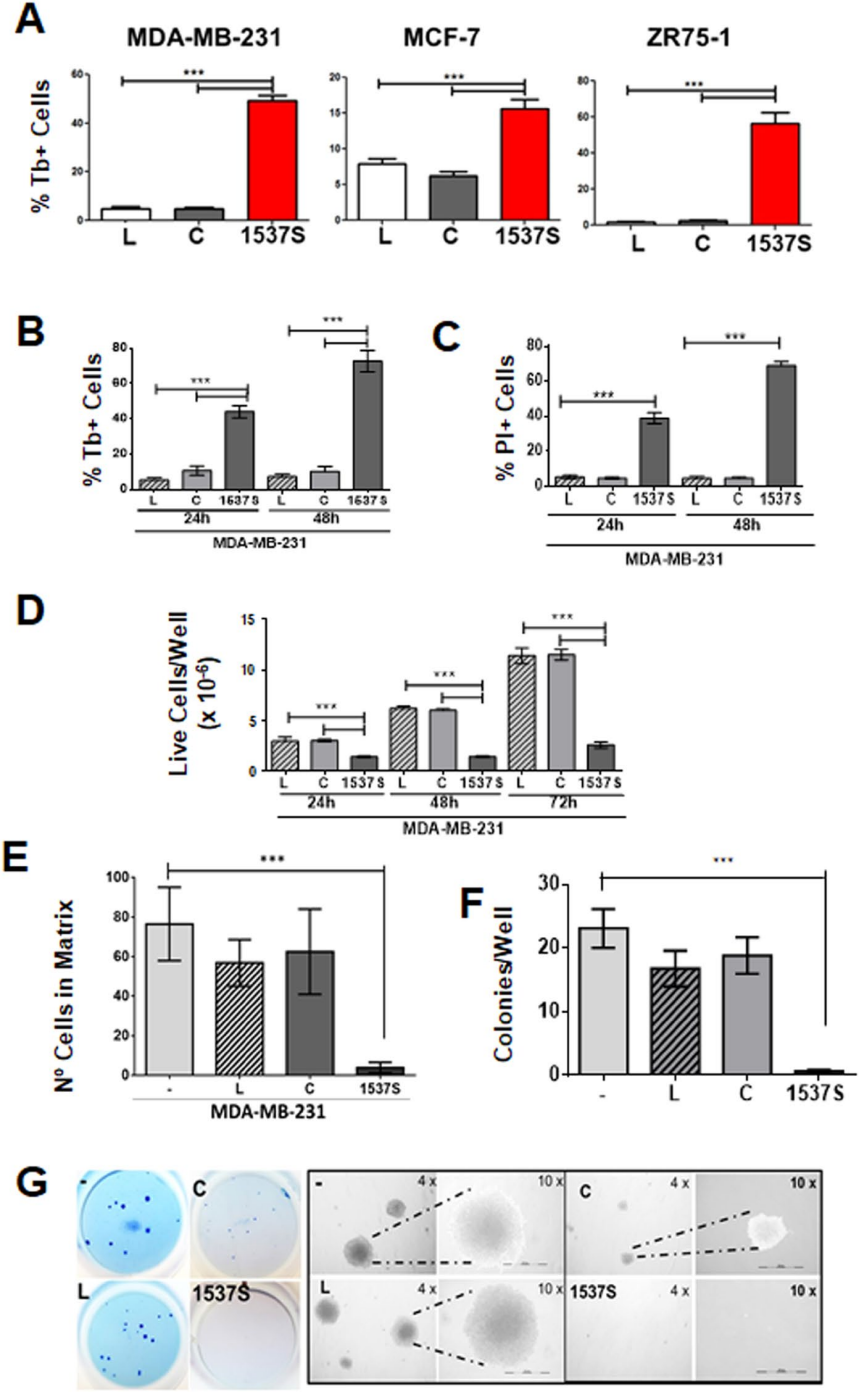
Knockdown of ASncmtRNA reduces viability and tumorigenic potential of human breast cancer cells. (**A)** MDA-MB-231, MCF7 and ZR-75-1 human breast cancer cells were transfected for 24 h with 200 nM ASO-1537S or ASO-C, or with transfection agent alone and cell death was measured by Trypan Blue (Tb) exclusion assay. (**B**,**C)** Death of MDA-MB-231 cells treated as in (**A**) for 24 and 48 h was determined by Tb (**B**) and propidium iodide (PI) (**C**) exclusion assays. (**D**) Live cells/well were evaluated by Tb exclusion after 24, 48 and 72 h. (**E)** MDA-MB-231 cells treated as in (A) were cultured in Matrigel-coated Boyden chamber inserts for 48 h. Inserts were fixed, stained with DAPI and nuclei were counted. (**F)** Anchorage-independent growth was evaluated in 12-well plates, in which 2 × 10^3^ Tb-negative MDA-MB-231 cells, transfected as in (**A**), were seeded onto soft agar. Colony formation capacity was evaluated after 21 days in culture. (**G)** Whole-well microphotographs of colonies and zoom-in under phase contrast microscopy at 4X and 10X magnification. All quantitative data shows average measurement from three independent experiments in triplicate. Statistically significant differences with respect to non-treated cells are indicated (**p < 0.01, ***p < 0.001). L, cells treated with Lipofectamine 2000; C, cells transfected with ASO-C and 1537 S, cells transfected with ASO-1537S.

Transfection of MDA-MB-231, MCF7 and ZR-75 cells with ASO-1537S (1537 S) for 24 h induced around 50%, 17% and 55% cell death respectively, while cells transfected with control ASO (C) or with Lipofectamine2000 transfection agent alone (L) displayed only a basal level of cell death (Fig. 1A). Among these three cell lines, MDA-MB-231 cells represent triple-negative breast cancer, the most aggressive breast cancer subtype and displays a high metastatic potential in *in vivo* models when compared to ZR-75 and MCF-7. Therefore, we focused our study on this cell line. Transfection efficiency in MDA-MB-231 cells reached 96% at 24 h (Supplemetary Fig. S1A). Viability was evaluated by Trypan blue (Tb) exclusion assay at 24 and 48 h, in which ASO-1537S-transfected cells displayed around 45 and 70%, respectively, while ASO-C-transfected cells and cells treated with transfection agent alone (L) only showed a basal level of cell death (Fig. 1B). Similar results were obtained with PI-stained cells subjected to flow cytometry (Fig. 1C). In addition, the remnant live cells from the ASO-1537 S treatment did not proliferate, in contrast to control cells (C and L) (Fig. 1D). The differences in death rates were not attributable to transfection efficiency since this parameter was very similar for both ASOs and over 90% (Supplementary Fig. S1B). After 48 h of transfection with ASO-1537 S, the remnant live cells displayed around 15-fold lower invasion capacity (Fig. 1E) and over a 10-fold lower anchorage-independent growth capacity, compared to controls (Fig. 1F,G), as evidenced by colony formation in soft agar.

### MDA-MB-231 cells release exosomes after ASncmtRNA knockdown

**Figure 2:**
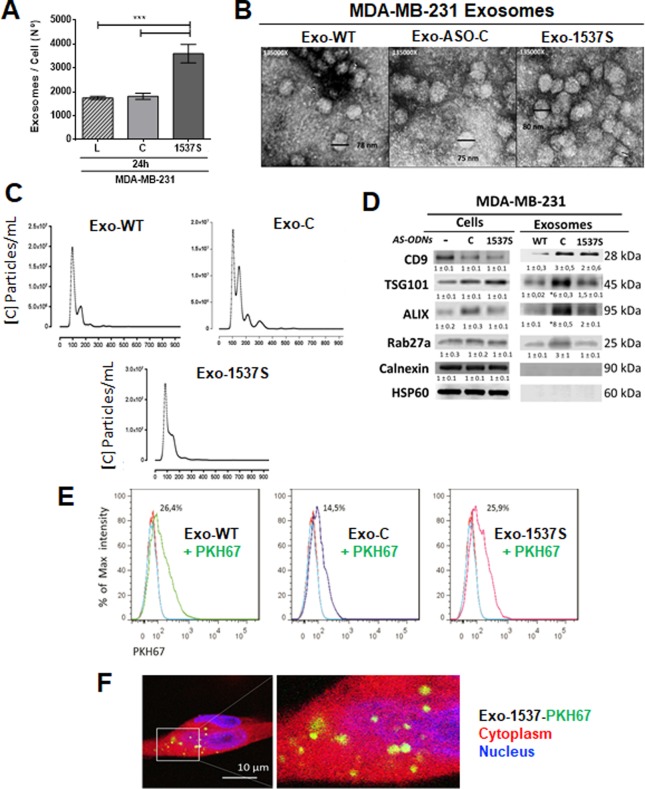
Characterization of Exo-WT, Exo-C and Exo-1537S exosomes and incorporation into MDA-MB-321 cells. MDA-MB-231 cells were transfected for 24 h as in Fig. 1 and exosomes were purified from the supernatants, obtaining 3 exosome preparations: Exo-WT (exosomes obtained from untreated cells), Exo-C (exosomes obtained from cells transfected with ASO-C) and Exo-1537 S (exosomes obtained from cells transfected with ASO-1537S). (**A)** Quantification of number of exosomes (NTA) purified from cells under the different treatments. Data are represented as average ± SEM (***p < 0,01, n = 3). (**B)** Exosome integrity of all samples was evaluated by Transmission Electron Microscopy (TEM) (135,000×). Bars indicate the diameter of some exosomes. (**C)** Exo-WT, Exo-C and Exo-1537 S samples were diluted so as to count 10 to 100 particles per record. The latter were then analyzed to determine the size with an approximation of the quantity of particles (concentration). The graph shows size distribution and number of particles in relation to diameter, with a mean diameter between 103 at 124 nm. (**D)** 30 µg of total exosome protein content of each sample was used to evaluate the classical exosome markers by Western blot: CD9, TSG101, ALIX and Rab27a. Calnexin and HSP60 were used as negative controls. A representative result for each blot is shown (n = 3). (**E)** Exosomes (Exo-WT, Exo-C and Exo-1537S) were labeled with the fluorescent dye PKH67 (green signal) and the number of exosomes was quantified by NTA. To perform the internalization protocol, labeled exosomes were added to MDA-MB-231 cells (1000 exosomes per cell), and then incubated for 3 h at either 37 °C or 4 °C. The internalization of Exo-WT, Exo-C and Exo-1537S exosomes into MDA-MB-231 cells was evaluated by fluorescence-activated cell sorting (FACS). Data was presented as percentage of signal intensity (n = 3) and was also visualized by confocal microscopy **(F)**, where the cytoplasm was stained with 2 µM CellTracker™ Red CMTPX (red signal), exosomes were labeled with PKH67 (green) and the nucleus was counterstained with DAPI (blue). A representative image of Exo-1537 S internalization into MDA-MB-231 cells is shown (magnification 100 × ).

We purified exosomes from MDA-MB-231 cells transfected with ASO-C (Exo-C), ASO-1537 S (Exo-1537S) or left untreated (Exo-WT) 24 h after transfection, when the percentage of apoptotic cells was approximately 50%. A higher proportion of exosomes per cell was obtained from ASO-1537S-treated cells (Fig. 2A). Purified vesicle integrity was confirmed by Transmission Electron Microscopy (TEM) (Fig. 2B) and expected particle size distribution was confirmed using Nanoparticle Tracking Analysis (NTA), showing exosome diameters ranging from 103 to 124 nm (Fig. 2C). Additionally, Western blot analysis confirmed the presence of the classical exosome markers CD9, TSG101, Alix and Rab27a in all exosome preparations. Calnexin and HSP60 were used to discard cell lysate contamination with proteins from the endoplasmic reticulum and mitochondria, respectively (Fig. 2D). These results show that the purified vesicles did not harbor cell lysate contaminants and were highly enriched in exosomes. To discard the presence of ASO-C or ASO-1537S in the exosomes, we transfected cells in the same manner described above, using Alexa fluor 488-labeled ASO-1537S or Cy3-labeled ASO-C, where we observed around 98% transfection efficiency (Supplementary Fig. S1B). Purified exosomes obtained after 24 h of transfection displayed no detectable fluorescence (Supplementary Fig. S1C). In addition, RT-PCR confirmed that exosome preparations were also devoid of ASncmtRNAs (Supplementary Fig. S2). These results were not unexpected since treatment with ASO-1537S induces knockdown of ASncmtRNAs^34,37,39^.

### Exo-WT, Exo-C and Exo-1537S exosomes released from transfected MDA-MB-231 cells are incorporated into wild type MDA-MB-231 cells

Exo-WT, Exo-C and Exo-1537S exosomes were loaded with the fluorescent dye PKH67 (green), washed to eliminate free dye, and quantified by NTA. MDA-MB-231 cells were incubated with the different exosomal preparations at a density of 1000 exosomes per cell. Internalization of labeled exosomes into MDA-MB-231 cells was measured by flow cytometry and confocal microscopy. The maximum percentage of intensity for the green signal in cells measured by flow cytometry was 26.4 ± 0.2%, 14.5 ± 0.8% and 25.9 ± 0.7% for MDA-MB-231 cells exposed to Exo-WT, Exo-C and Exo-1537S, respectively (Fig. 2E). Additionally, the presence of exosomes (green signal) was readily observed in the cytoplasm (red signal) of recipient cells by confocal microscopy (Fig. 2F). Since there was no difference in the intensity of fluorescent signals, the incorporation of exosomes into cells was successfully achieved using all three exosome preparations.

### Exo-1537S decrease proliferation of MDA-MB-231 cells

**Figure 3:**
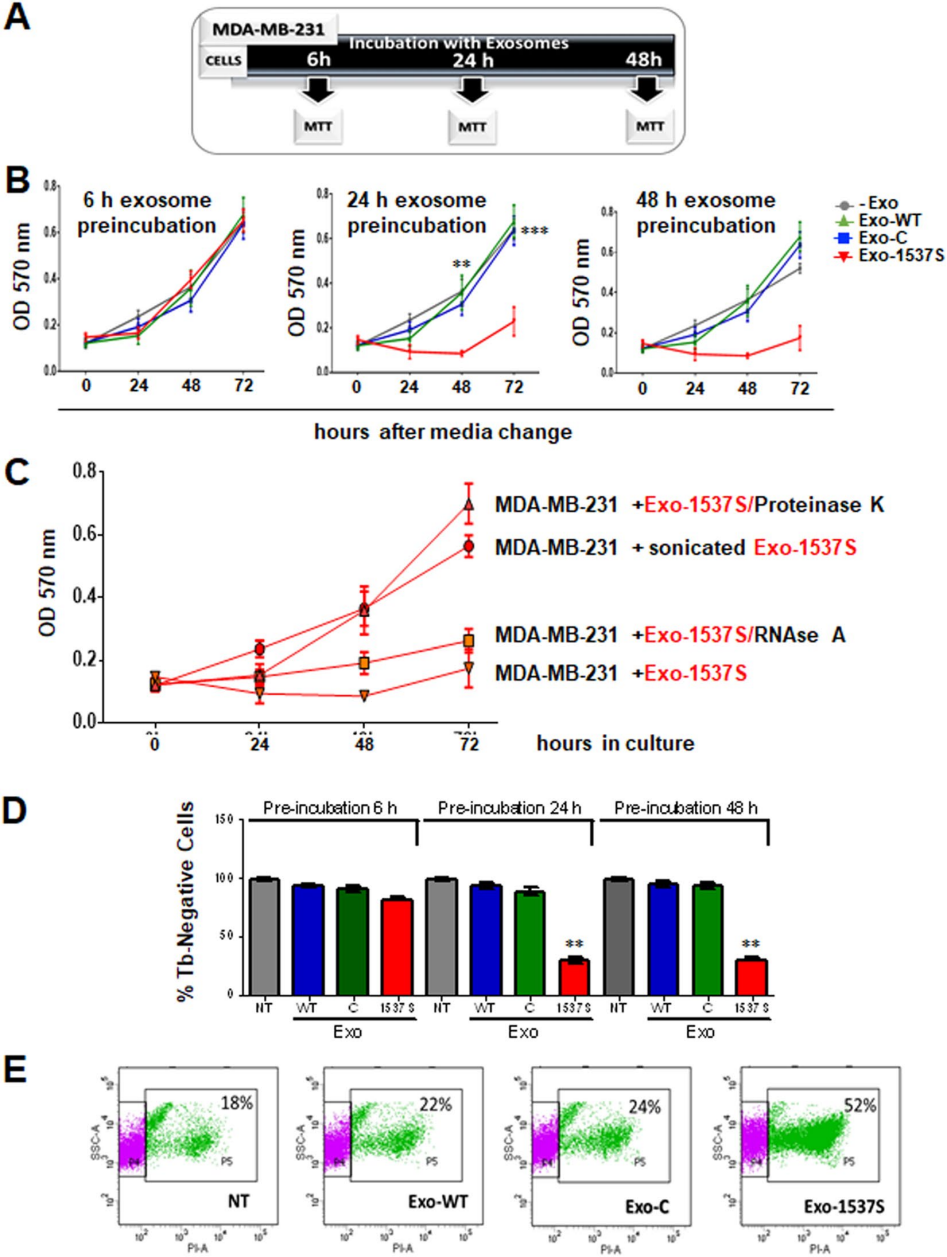
Exo-1537S decrease viability of MDA-MB-231 cells. (**A)** Diagram of the different incubation periods of MDA-MB-231 cells with exosomes. MDA-MB-231 cells were incubated alone or in the presence of Exo-WT, Exo-C or Exo-1537 S (10 μg) for 6 h **(B)**, 24 h **(C)** or 48 h **(D)** and proliferation was measured by MTT after switching to fresh medium. (**E)** Exo-1537S exosomes (10 μg) were treated with RNaseA or proteinase K, sonicated (5 min) or left untreated, after which they were added to MDA-MB-231 cells. Viability was determined by MTT assay at 24, 48 and 72 h. (**F)** MDA-MB-231 cells were pre-incubated for 6, 24 or 48 h alone or in the presence of Exo-WT, Exo-C or Exo-1537S exosomes. After switching to exosome-free fresh media, cells were further cultured for 48 h and viability was determined by Tb exclusion assay. (**G)** MDA-MB-231 cells were pre-incubated for 24 h either alone or in the presence of Exo-WT, Exo-C or Exo-1537S exosomes, followed by 48 h of culture in fresh media. Viability was determined by PI exclusion and flow cytometry analysis. The data correspond to the average of 3 independent experiments in triplicate. Statistically significant differences are indicated compared to wild-type cells (**p < 0.01).

MDA-MB-231 cells were incubated during different time points (6, 24 and 48 h) with exosomes (equivalent to 1 µg/mL total protein). After incubation, exosome-containing media was replaced by fresh culture media and proliferation was determined by MTT assay at 24, 48 and 72 h after media change (Fig. 3A). After 6 h of exosome incubation, there was no difference in cell proliferation (Fig. 3B). However, cells incubated for 24 h and 48 h with Exo-1537S decreased at least 3 times their proliferation rate compared to untreated cells or cells exposed to Exo-WT or Exo-C (Fig. 3B). Cell proliferation decreased to a minimum at 48 h, after which proliferation showed only a modest recovery.

In order to assess whether this effect in proliferation is related to the inner content of the exosomes, we pre-treated Exo-1537S with proteinase K, RNase A or sonication before adding them to MDA-MB-231 cells. As shown in Fig. 3C, RNase A pre-treatment did not reverse the antiproliferative effect of Exo-1537S, suggesting that putative RNAs that could be adhered to the outer surface of the exosomes do not play a role in this process. In contrast, and as expected, sonication of Exo-1537S caused a loss in the anti-proliferative effect of these exosomes. Moreover, proteinase K treatment also reversed the anti-proliferative activity of these exosomes, suggesting that external or transmembrane proteins present in the membranes of exosomes are responsible for the lower proliferation rates observed. To determine the effect of exosomes on viability, MDA-MB-231 cells were incubated with the exosomal preparations for 6, 24 or 48 h, after which exosome-containing media was removed and cells were further cultured in freh media for 48 h. Cell death was assessed by Tb exclusion assay. A strong decrease in the percentage of viable cells was observed when incubated with Exo-1537S for 24 or 48 h, compared to controls (Fig. 3D). Similar results were obtained by PI staining followed by flow cytometry of cells treated for 24 h with exosomal preparations or left untreated and then cultured for 48 h in fresh media. The results showed that Exo-1537S treated cells displayed an average proportion of 52% PI-positive cells, compared to 18, 22 and 24% in controls (Fig. 3E).

### Exo-1537S decreases invasive capacity, spreading and colony formation of breast cancer cells

**Figure 4:**
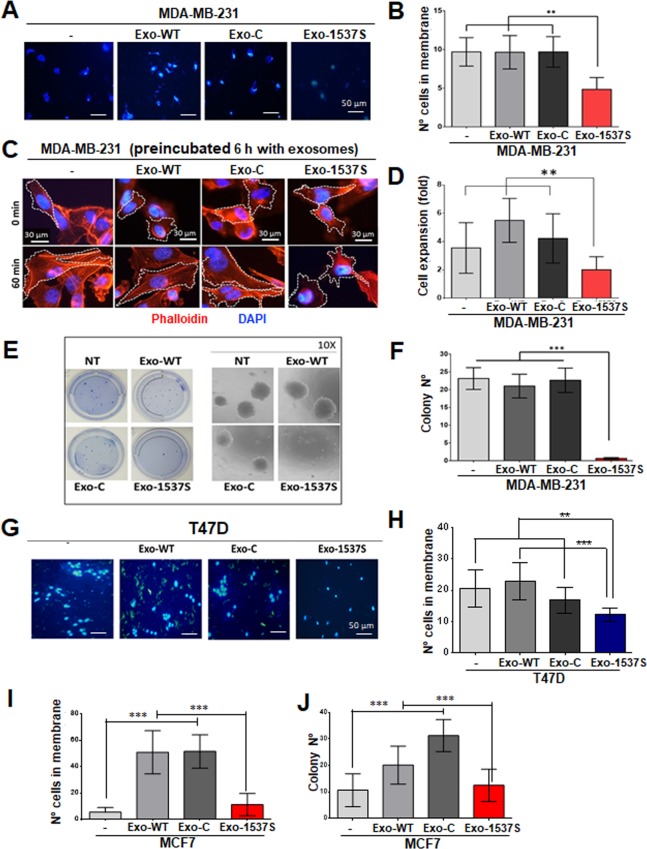
Exo-1537S exosomes decrease invasion, spreading and anchorage-independent growth of MDA-MB-231 cells. **(A)** MDA-MB-231 cells were seeded into Matrigel-coated Boyden chamber inserts, either alone (−) or in the presence of 10 µg Exo-WT, Exo-C or Exo-1537S exosomes. After 48 h, inserts were fixed, stained with DAPI and nuclei in membranes were counted. A triplicate analysis **(B)** revealed a two-fold reduction in invasion in cells treated with Exo-1537S. **(C)** MDA-MB-231 cells were treated as in (**A**) for 6 h and then allowed to attach and spread onto fibronectin-coated coverslips for 60 min. Cells were stained with phalloidin–Rhodamine and DAPI and analyzed by fluorescence microscopy. At least 10 images per condition were analyzed (5–10 cells per image). Cell area was determined using the imageJ software. **(D)** Cell spreading was plotted in terms of increased size of NT (−) compared to Exo-WT, Exo-C and Exo-1537S. **(E)** MDA-MB-231 cells were grown alone or in the presence of 10 µg Exo-WT, Exo-C or Exo-1537S for 24 h, after which they were seeded onto soft agar. Colonies were scored at 21 days. A zoom-in of colonies for each sample is shown on the right. A triplicate analysis **(F)** shows an almost complete abolition of colony formation capacity in cells treated with Exo-1537S. Statistical significance compared to wild-type cells (−) are indicated (**p < 0.01, ***p < 0.001, n = 3). **G)** T47D cells were seeded into Matrigel-coated Boyden chamber inserts, either alone (−) or in the presence of 10 µg Exo-WT, Exo-C or Exo-1537 S exosomes. After 48 h, inserts were fixed, stained with DAPI and nuclei in membranes were counted. (**H)** The quantification of number of cell in membrane. (**I)** MCF7 cells were evaluated in invasion assays like MDA-MB-231 and T47D. **J)** MCF7 cells were grown alone or in the presence of 10 µg Exo-WT, Exo-C or Exo-1537S for 24 h, after which they were seeded onto soft agar. Colonies were scored at 21 days (***p < 0.001, n = 3).

Considering that incubation of MDA-MB-231 cells with Exo-1537S for 6 h did not affect their proliferation rate, (Fig. 3B), we evaluated whether other tumorigenic capacities were altered. MDA-MB-231 cells were pre-incubated with exosomes for 6 h, and cells were further cultured for 48 h. Cells were then collected and used to determine invasion for 48 h. We observed a two-fold decrease in invasion capacity in cells exposed to Exo-1537S vesicles compared to cells exposed to control vesicles (Fig. 4A,B). Of note, untreated cells and cells exposed to Exo-WT or Exo-C did not display any differences between each other in terms of invasion capacity (Fig. 4A,B). To evaluate the movement of cells in a matrix, we measured cell spreading on fibronectin-coated coverslips after being exposed to exosomes for 6 h. Spreading decreased at least 2-fold in cells exposed to Exo-1537S vesicles compared to cells exposed to Exo-WT vesicles (Fig. 4C,D). We also evaluated the anchorage-independent growth ability of MDA-MB-231 cells exposed to Exo-1537S, by colony formation in soft agar. Cells were pre-incubated with 10 µg (protein content) of exosomes for 24 h, after which cells were seeded in soft agar and colonies > 100 µm in diameter were scored at 21 days. Exo-1537S vesicles almost completely suppressed clone formation compared to cells incubated with control vesicles (Fig. 4E,F). For comparison, we evaluated the effects of Exo-1537S on invasiveness of two less aggressive human breast cancer cell lines, T47D and MCF7, exposed to exosomes. Invasion capacity of T47D cells exposed to Exo-1537S showed a significant decrease compared to controls, albeit modest when compared to MDA-MB-231 cells (Fig. G, H). In MCF7 cells, an increase in invasion potential of about 5 times was observed when incubated with Exo-WT and Exo-C exosomes, compared to untreated cells (Fig. 4I), suggesting that the content from those exosomes potentiated the tumorigenic property of this cell line. However, Exo-1537S incubation was able to reverse this effect (Fig. 4I). Similar results were obtained for colony formation in MCF7 cells, in which incubation with Exo-WT or Exo-C enhanced anchorage-independent growth, but Exo-1537S reversed this effect (Fig. 4J).

### Tumorigenesis of MDA-MB-231 cells in an *in vivo* breast cancer carcinomatosis model is enhanced by Exo-WT and Exo-C and decreased by Exo-1537

**Figure 5:**
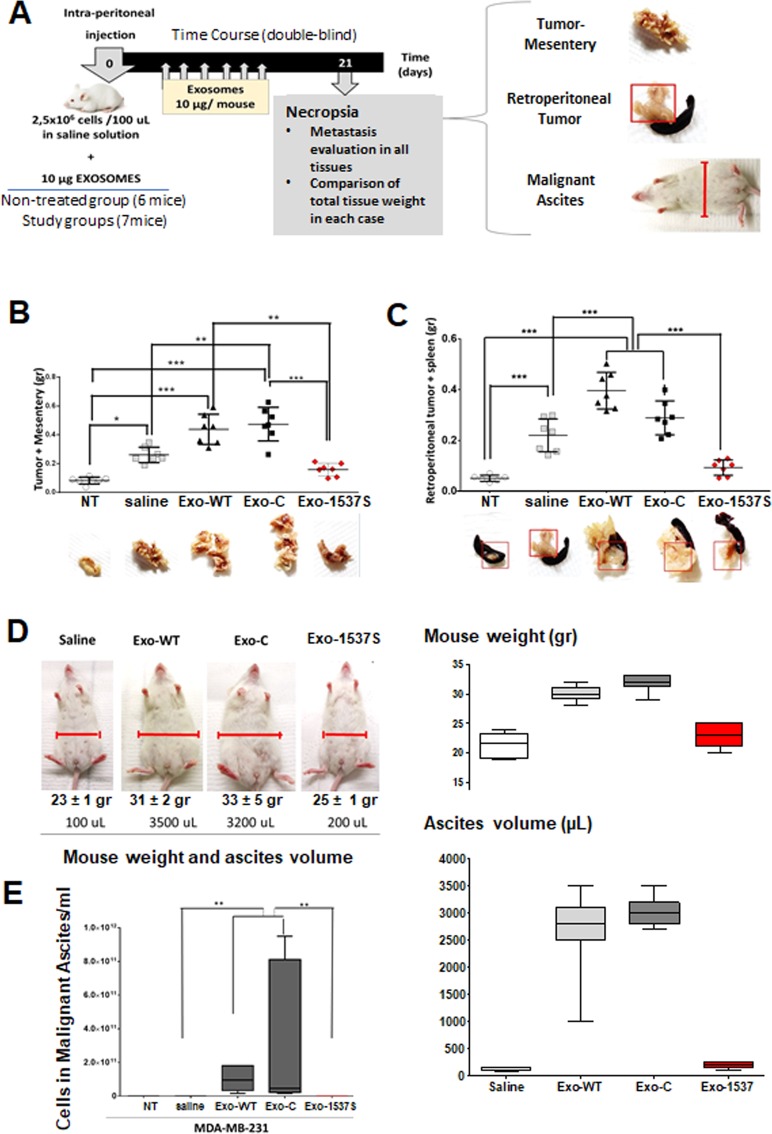
Exo-WT and Exo-C increase and Exo-1537S decrease tumorigenic capacity in an *in vivo* breast cancer model of peritoneal carcinomatosis. (**A**) BalbC NOD/SCID mice were injected ip with 2,5 × 10^6^ MDA-MB-231 cells in saline alone (250 µl) or containing 10 µg Exo-WT, Exo-C or Exo-1537S (7 mice per group). A separate control group of 6 mice was left uninoculated. Every three days, the animals were injected ip with 10 µg of the corresponding exosome preparation, or saline and mice were euthanized on day 21 post-inoculation. The rating of metastasis was evaluated in tumor-mesentery, spleen and by formation of malignant ascites. (**B)** Total weight of tumor-mesenteric tissue. (**C)** Total weight of spleen/pancreas. Statistically significant differences are indicated *p < 0.05, **p < 0.01, ***p < 0.001. Photographs of representative experiments showing tumor growth in these tissues are displayed in the lower part of each graph. (**D)** Photographs of representative mice from each group showing comparison in abdominal width, total mouse weight and ascites volume. (**E)** Quantification of cells/ml in ascitic fluid. Statistically significant differences are indicated *p < 0.05, **p < 0.01, ***p < 0.001, Tukey Multicomparison assays.

Three groups of 7 BalbC NOD/SCID mice, 5–7 weeks of age, were injected intraperitoneally (ip) with 2.5 × 10^6^ MDA-MB-231 cells, together with Exo-WT, Exo-C or Exo-1537S (10 μg per mouse). A separate control group of 7 mice was inoculated with cells + saline only and another group of 6 mice was left uninoculated (NT). Injections were performed in a blinded fashion. At 21 days, all animals were sacrificed under anesthesia and tumor/mesentery mass, retroperitoneal tumor and malignant ascites were collected (Fig. 5A). Solid tissues were fixed and weighed. The average total tumor/mesenteric mass (g) in mice inoculated with Exo-WT (0.44 ± 0.004 g) and Exo-C (0.47 ± 0.006 g) was significantly higher than in mice injected with cells only (saline; 0.26 ± 0.0062 g). In striking contrast, Exo-1537S co-injection inhibited tumor growth compared to the saline group, reaching an average of 0.16 ± 0.003 g and showed a mesenterial mass slightly higher than uninoculated mice (NT; 0.07 ± 0.003 g) (Fig. 5B). Conclusively, Exo-WT and Exo-C-treated mice exhibited average tumor sizes of 0.37 and 0.4 g, respectively, corresponding to approximately twice the size of mice inoculated with cells alone (0.19 g). In contrast, Exo-1537S-treated mice displayed tumors that were, on the average, half the mass of the saline group (0.09 g), showing that, *in vivo*, these exosomes also inhibit MDA-MB-231 cell proliferation.

Similar findings were observed for retroperitoneal tumor/spleen mass. The mice that received Exo-WT and Exo-C showed a total average mass of 0.40 ± 0.05 and 0.29 ± 0.06 g, respectively, compared to mice injected with cells only (saline; 0.22 ± 0.06 g). Exo-1537 S-treated mice, on the other hand, showed an average total mass of 0.087 ± 0.01, again only slightly higher than uninoculated mice (0.051 ± 0.005 g) (Fig. 5C). This means that control exosomes, Exo-WT and Exo-C, augmented the retroperitoneal tumor mass 2- and 1.4-fold, respectively, while Exo-1537S decreased tumor mass approximately 4.7-fold, compared to the saline group.

Development of malignant ascites was only observed in mice treated with Exo-WT and Exo-C (9,6 × 10^10^ and 3,4 × 10^11^ cells/ml, respectively) (Fig. 5D), while in the saline group, the number of cells found in the abdominal cavity was lower than 180.000 cells/mL (Fig. 5E).

### MDA-MB-231 cells release exosomes with differential molecular content after ASncmtRNA knockdown

**Figure 6:**
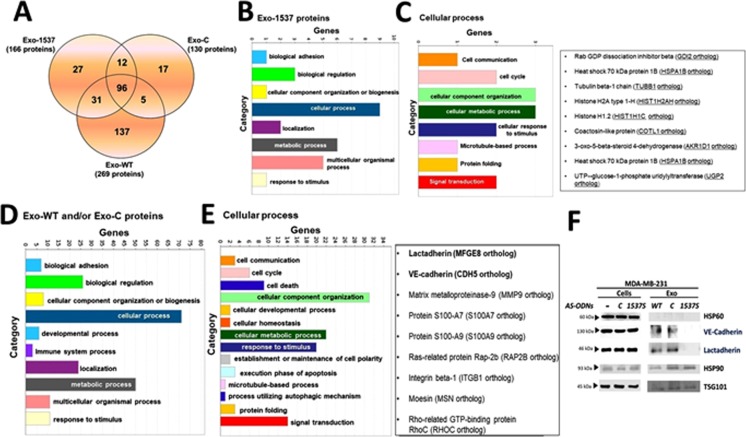
Mass spectrometry and pathway analysis of Exo-WT, Exo-C and Exo-1537S proteins. (**A**) Venn diagram of proteins identified in exosomal preparations. (**B)** Gene ontology (GO) enrichment analysis of proteins only found in Exo-1537S (27 proteins) in which the top 8 significantly-enriched GO categories are indicated under “Cellular process”. (**C)** Gene ontology (GO) enrichment analysis of Exo-1537S proteins in which the 8 significantly enriched GO categories under “Cellular process” (depicted in B) are indicated (left panel). The list (right panel) shows several protein candidates representing each enriched category. (**D)** Gene ontology (GO) enrichment analysis of Exo-WT and/or Exo-C proteins (159 proteins) showing the top 10 significantly-enriched GO categories under “Cellular process”. (**E)** Gene ontology (GO) enrichment analysis of Exo-WT and/or Exo-C proteins showing the 14 significantly enriched GO categories under “Cellular process” (depicted in D). The list (right panel) shows several protein candidates associated to cancer progression identified in each category. (**F)** Western blot analysis of invasion-promoting protein candidates VE-Cadherin and Lactadherin, obtained from the proteomic analysis. Results from a representative experiment are shown. Hsp60 was included as a negative control; Hsp90 and Tsg101 were used as positive markers (n = 3).

A strong decrease of tumorigenic properties in MDA-MB-231 cells is observed after incubation with Exo-1537 S vesicles and, conversely, an increase in these properties is attained with Exo-WT and Exo-C vesicles. Therefore, we sought to determine which components of the molecular cargo of the extracellular vesicles are responsible for these effects. For this purpose, we performed a proteomic analysis of each exosome preparation. A Venn diagram of the results shows that 137 proteins are unique to Exo-WT vesicles, 17 proteins are exclusive for Exo-C and 27 proteins are unique for Exo-1537S vesicles (Fig. 6A). The latter contained at least 6 subunits of the proteasome complex and mostly proteins associated to the actin cytoskeleton (Supplementary Material Mass Spectrometry Analysis). Comparison among all three exosome pools yielded 96 proteins in common, in which the majority corresponded to histones, complement system proteins, Fibrinongen, Lactoferrin and Apolipoproteins (Supplementary Material Mass Spectrometry Analysis). Five proteins were shared only by Exo-WT and Exo-C (Fig. 6A). To gain insight into signaling pathways that might be controlled by the proteins enriched in Exo-WT, Exo-C and Exo-1537S vesicles, we performed a GO analysis (PANTHER) and detailed pathway analysis proteins obtained by using a Reactome platform. The Gene Ontology (GO) enrichment analysis of Exo-1537S proteins only found in this sample (27 proteins) is depicted in Fig. 6B, showing the 8 most significantly enriched gene categories. We then performed GO enrichment analysis of the subgroup of genes categorized under “Cellular process”, showing the 8 most-enriched gene categories among this group (Fig. 6C, left panel). The results showed several protein candidates representing each enriched category: RabGDP, HSP70, Tubulin Beta 1 Chain, Histone H2A type 1-H, Histone H1.2, Coactosin-like protein, 3-oxo-5-beta-steroid 4-dehydrogenase and UTP-glucose-1-phosphate uridyliltransferase (Fig. 6C, right panel). Likewise, we carried out a GO enrichment analysis of the 159 proteins found only in Exo-WT and/or Exo-C preparations (Fig. 6A), where we observed 10 significantly enriched gene categories (Fig. 6D). Further GO analysis of the genes categorized under “Cellular process” rendered 14 enriched categories (Fig. 6E, left panel). Among these proteins we found Lactadherin, VE-cadherin (Cadherin-5), MMP9, S100A7, S100A9, Ras-related protein Rap-2b, Integrin β-1, Moesin and Rho-related GTP-binding protein RhoC (Fig. 6E, right panel).

In summary, the proteomic analysis allowed us to determine the presence of proteins contained differentially in Exo-1537, compared to the controls, Exo-C and Exo-WT. In the latter two preparations, we detected the presence of VE-Cadherin (CDH5) and Lactadherin (MFG-E8) and several members of the S100 family of proteins reported to participate in organotropic breast cancer metastasis^40^. In order to corroborate these findings, we performed Western blot analysis, in which we detected the presence of the invasion-promoting protein candidates VE-Cadherin and Lactadherin (Fig. 6F), but the other candidates assayed did not display differential expression between controls (Exo-WT and Exo-C) and Exo-1537S particles (Fig. 6F). The levels of all the proteins analyzed showed no difference in total cell lysates (Fig. 6F), confirming that the exosome treatments did not alter the expression of these protein genes.

## Discussion

ASncmtRNA knockdown causes significant cell death in a wide array of cancer cell lines^35–37,41^. The percentage of cell death varies among cell lines^35^ and, as our results show, this percentage is around 16–40% in several breast cancer cells at 24 h (Fig. 1A). The question that arises is whether the remaining live cells at 24 h post-transfection preserve their tumorigenic potential or lose these traits upon treatment. As shown in Fig. 1E-G, after ASncmtRNA knockdown remnant cells drastically reduce their invasive and anchorage-independent growth capacities. The mechanisms underlying the effects of ASncmtRNA knockdown on tumor cells is not completely understood. A proposed mechanism involves the emergence of miRNAs that target survival factors such as survivin, thus triggering apoptosis^35^. In the present study we aimed at a different approach, which was to isolate extracellular vesicles that are released after ASncmtRNA knockdown at 24 h post-transfection of MDA-MB-231 breast cancer cells (where we found approximately 60% live cells), since these vesicles might contain molecules (proteins or RNAs) which could affect target cells. Exosomes purified post-knockdown of ASncmtRNA displayed similar size and distribution as those isolated from ASO-C-treated and untreated cells (Fig. 2B,C). Detection of the exosome markers ALIX, TSG101, CD9 and Rab27a^42^ confirmed the exosomal nature of the vesicles (Fig. 2D). Thus, in the three experimental conditions, exosomes are similar in appearance but may differ in their content and in their behavior.

Proliferative rate of tumor cells is highly related to metastasis^42,43^. Accordingly, an incubation time of 24 h or more with exosomes modulates MDA-MB-231 tumor cell proliferation (Fig. 3B). External treatment of Exo-1537S with RNase A prior to incubation with cells exerts no effect on the antiproliferative activity of these vesicles (Fig. 3C), excluding the possibility that the observed effects are due to RNAs adhered to the outer side of their membranes. Sonication, on the other hand, precluded said effect of these exosomes (Fig. 3C), suggesting that integrity of the vesicles is essential for their activity. External treatment with proteinase K also affected their ability to block proliferation (Fig. 3C), suggesting the presence of surface or transmembrane proteins which could be directly involved in the effects on proliferation or in docking and internalization of exosomes. Altogether, these results suggest that the effects of Exo-1537S vesicles on proliferation of target cells are due to their cargo, which could be proteins and/or RNAs.

Tumorigenic properties of MDA-MB-231 cells such as migration, invasion, spreading and anchorage-independent growth are also diminished in the presence of Exo-1537S (Fig. 4). Each of these characteristics are part of different stages of metastasis^44^. Our results suggest that the first metastatic behavior affected is migration and consequently spreading, since a 6 h incubation of cells with Exo-1537S was sufficient to visualize a decrease in these properties (Fig. 4A–D). Proliferation and anchorage-independent growth are affected after 24 h of incubation with Exo-1537S and invasion is decreased only after 48 h of exosome incubation. On the other hand, our results in a murine xenograft peritoneal carcinomatosis model showed that Exo-1537S injection, in conjunction with MDA-MB-231 cells, displayed a strong reduction in tumor growth compared to Exo-WT and Exo-C and, furthermore, reduced the size of mesenteric and retroperitoneal tumor mass, even compared to cells injected alone (Fig. 5B,C), suggesting that the molecular cargo of Exo-1537S contains factors that inhibit tumorigenesis. Furthermore, Exo-WT and Exo-C vesicles promoted tumor growth beyond that of cells-only-injected mice (Fig. 5B,C). These *in vivo* results strongly suggest that exosomes released from control cells contain factors that enhance tumorigenic properties, while exosomes secreted upon ASncmtRNA knockdown harbor factors that promote the opposite outcome, similar to the effects brought upon directly by transfection of a myriad of tumor cell types with ASO-1537S^34,37,39^.

Exo-WT and Exo-C vesicles enhanced invasiveness and anchorage-independent growth potential of MCF7 cells, a cell line of lower aggressiveness than MDA-MB-231, whilst Exo-1537S vesicles revert this effect (Fig. 4I,J). Furthermore, T47D cells, another human breat cancer cell line, undergo a significant decrease in their invasion capacity in the presence of Exo-1537 S vesicles (Fig. 4G,H). It has been reported that breast cancer cells could increase their metastatic potential by receiving exosomes secreted by metastatic cell lines^45^, although this report does not describe which proteins might be participating in this behavior. Exosomes trigger these effects by delivering their vesicle content to recipient cells in a paracrine or autocrine manner^46^. Nevertheless, there are very few studies regarding the effects of exosome content in breast cancer cells^47^, even though it has been suggested that they could be potential tumour and metastasis markers in this cancer type^47,48^.

A recent report showed that breast cancer exosomes enriched in α6β4 and α6β1 integrins were able to promote lung-specific metastasis^47^. On the other hand, αvβ5 integrin in exosomes was responsible for promotion of liver metastasis. This research does not discard micrometastasis to other tissues or the fact that this type of homing may be exclusive of integrins or members of the S100 family of proteins collaborating in targeting certain organs for metastasis^49–51^. It should be noted that the authors also showed that metastasis is not a random process^47,48^.

Our proteomic analysis shows that Exo-WT and Exo-C contain a set of proteins that are absent from Exo-1537S (Fig. 6). Among these proteins, we focused on VE-Cadherin and Lactadherin, which have been suggested to play a role in the increase of tumorigenic properties^52–56^. We corroborated this data with Western blot analysis, which showed the differences in the expression of these two proteins comparing the control vesicles (Exo-WT and Exo-C) with Exo-1537 S (Fig. 6).

VE-Cadherin is a well-known factor for increased vascular permeability in estrogen-positive breast cancer^57^ and its expression correlates with poor survival prognosis in human gastric cancer and can therefore be used as a biomarker^58,59^. VE-Cadherin expression has been related to melanoma^60^ and breast cancer progression^61^. Nevertheless, the presence of VE-Cadherin in exosomes or the possible effects that these vesicles may have in the tumorigenic potential of breast cancer cells has not been previously determined. Lactadherin, on the other hand, regulates Cyclin D1 and D3 expression, leading to an increase in tumorigenic potential of epithelial breast cancer cells^54^. These evidences support our results on promotion of aggressiveness of a non-metastatic cell line such as MCF7, in terms of enhancement of migration, invasion and anchorage-independent growth of recipient cells^45^. This type of event could probably favor the communication among tumor cells of different aggressiveness within the tumor itself and therefore promote the progression of the disease.

Proteomic analysis showed the absence of Lactadherin and VE-Cadherin and enrichment of proteasome subunits in Exo-1537S, demonstrating the modulation of protein exosome cargo after ASncmtRNA knockdown. These changes in exosome cargo could negatively affect the preparation of breast cancer metastatic niches^28^. To our knowledge, this is the first report that describes the presence of two different known tumor-promoting cadherins in exosomes, suggesting a possible role of these cadherins in metastatic niche preparation. Several studies have demonstrated the role of exosomes in the promotion of metastasis, depending on their cargo^62,63^. Our *in vivo* results point to the same direction, showing increase in tumor mass and metastatic sites, together with increased ascites accumulation in animals that received Lactadherin and VE-cadherin-containing Exo-WT and Exo-C in comparison to Exo-1537S recipient animals. These results suggest two possible roles of exosomal Lactadherin in the metastatic process: first, as an “adherence promotor” for the incorporation of exosome cargo into recipient cells^43^; and second, as an “effector molecule” itself, together with VE-cadherin (among others). This could result in the modulation of different pro-metastatic processes in animal models, such as increasing vascular permeability and therefore promoting extravasation, increasing tumor cell adhesion and/or transforming the phenotype of healthy cells, granting them tumorigenic properties (Supplementary Fig. S3). To date, there are no functional studies showing the participation of Lactadherin and VE-cadherin as promoters of tumorigenic capacities. Therefore, taken together, this study shows for the first time the modulation of exosome cargo derived from breast cancer cells after ASO therapy against ASncmtRNA. This therapy, along with the direct induction of cell death, abolishes the release of exosomes with pro-metastatic activity from breast cancer cells, decreasing the amount of proteins in the exosome cargo which can participate in the metastatic cascade such as Lactadherin and VE-cadherin. Further studies are under way, in order to determine if these proteins present in exosomes are responsible for the effects observed in recipient cells.

## Methods

The experimental protocols were approved by appropriate guidelines of Biosecurity Committee of Fundación Ciencia & Vida, and no including any relevant details.

### Cell culture

The human breast cancer cell line MDA-MB-231 (pleural fluid-derived metastatic adenocarcinoma) was purchased from ATCC (HTB-26), and cultured in DMEM F12 (Gibco). ZR-75, T47-D and MCF-7 cell lines were purchased from ATCC, and were cultured in RPMI (Gibco). All media was supplemented with 10% fetal bovine serum (FBS, Hyclone), 10,000 U/mL penicillin, 10 mg/mL streptomycin sulfate and 25 mg/mL amphotericin B and cells were cultured at 37 °C with 5% CO_2_. Prior to use, FBS was depleted of extracellular vesicles (Exo-Free FBS) by ultra-centrifugation at 100,000 × *g* for 16 h at 4 °C. The supernatant was then filtered through 0.2 µm syringe filters (GE Healthcare).

### Cell transfection

Antisense oligonucleotides (ASOs) used in this study were synthesized by Integrated DNA Technologies with 100% phosphorothioate (PS) linkages. The sequences of the ASOs were 5′-CACCCACCCAAGAACAGG-3′ (ASO-1537S) and 5′-AGGTGGAGTGGATTGGGG-3′ (control ASO; ASO-C). In order to assess transfection efficiency, cells were transfected with ASOs labeled at the 5′ end with Alexa Fluor 488. For exosome preparation, 10^6^ MDA-MB-231 cells were seeded into 10 mm Petri dishes (Nunc) and transfected after 24 h with 200 nM ASO and 2 μg/ml Lipofectamine2000 (Invitrogen). After 6 h, cells were washed 3 times with PBS, Exo-free FBS-containing complete media was added and cells were further incubated for 24 h.

### Cell viability

Cells transfected as above were harvested at 24 or 48 h post-transfection and total cell number and viability was determined by Trypan blue (Tb) or propidium iodide (PI) exclusion, as previously described^35^. Flow cytometry was performed on a BD-FACS Canto II Flow Cytometer (Fundación Ciencia & Vida).

### Exosome isolation

Exosomes were isolated from culture supernatants of cells transfected with ASO-C (Exo-C), ASO-1537S (Exo-1537S) and untreated cells (Exo-WT), as described above. Culture media (200 ml) was subjected to an initial centrifugation at 300 × *g* for 10 min to remove whole cells and cell debris. Supernatant was centrifuged at 16,000 × *g* for 30 min at 4 °C. Subsequently, exosomes were isolated using the Exospin^TM^ system (Cell Guidance Systems), according to manufacturer’s guidelines.

### Transmission electron microscopy (TEM)

Exosomes were suspended in ice-cold PBS containing 2% *p*-formaldehyde and mounted on copper grids, fixed in ice-cold PBS/1% glutaraldehyde for 5 minutes and washed in sterile distilled water. Grids were then contrasted with 4% uranyl acetate solution at pH 7 for 5 min and embedded in methyl cellulose-UA for 10 min on ice. Images were acquired on a Philips Tecnai 12 BioTwin transmission electron microscope (Pontificia Universidad Católica de Chile), operating at 80 kV.

### Nanoparticle tracking analysis (NTA)

The NanoSight NS300 (NanoSight NTA 2.3 Nanoparticle Tracking and Analysis Release Version Build 0033) (Malvern Instruments) was used to measure concentration and size of particles. Samples were diluted 1:200 in PBS and the camera was set to capture 3 videos per sample with 30 seconds of duration per video. The videos were then analyzed to determine the size distribution with an approximation to the quantity of particles.

### RT-PCR amplification

Total RNA from the lysate of MDA-MB-231 cells or MDA-MB-231 cells treated with wild type (Exo-WT), ASO-control (Exo-C) and ASO-1537S (Exo-1537) exosomes were extracted using TriZOL (Ambion) as described elsewhere^1,2^. RNA preparations were treated with 2 U of TURBO DNA-free (Ambion) according to manufacturer’s instructions. Reverse transcription was carried out with 100 ng freshly prepared RNA, 50 ng random hexamers, 20 U RNase OUT (ThermoFisher Scientific), 0.2 mM dNTPs and 200 U M-MLV reverse transcriptase (Invitrogen) in a final volume of 20 µl. Two µl cDNA were used to amplify ASncmtRNA-2 by PCR reaction in a reaction mix containing 2.5 U GoTaq (Promega), 1.5 mM MgCl_2_, 0.4 µM each dNTP and 0.2 µM of each primer in a final volumen of 50 µl. The amplification program used was: 100 °C for 10 min, 70 °C for 10 min, 80 °C for 10 min and 26 cycles consisting of 94 °C for 1 min, 58 °C for 1 min and 72 °C for 1 min. The primer sequences for amplification of ASncmtRNA-2.were: 5′ACCGTGCAAAGGTAGCATAATCA (forw) and 5′CAAGAACAGGGTTTGTTAGG (rev).

### Western blot

Cells were detached by trypsinization and lysed in 0.2 mM HEPES (pH 7.4) containing 0.1% SDS, 1 mM Na_3_VO_4_ (phosphatase inhibitor) and protease inhibitor cocktail (10 µg/mL benzamidine, 2 µg/mL antipain, 1 µg/mL leupeptin and 1 mM PMSF). Purified exosomes were sonicated in 0.9% NaCl containing 1 mM PMSF. Protein concentration was quantified with the Bradford microplate system Gen5TM EPOCH (BioTek). Total protein extracts (30 µg/lane) were resolved by SDS-polyacrylamide gel electrophoresis (SDS-PAGE) and transferred to polyvinylidene difluoride membranes (PVDF). Membranes were blocked with 5% skim milk in PBS/0.1% Tween (PBST) and then probed with antibodies against CD9 (m-monoclonal; Santa Cruz; 1:200), TSG101 (m-monoclonal; Santa Cruz; 1:200), ALIX (m-monoclonal; Cell Signaling; 1:500), Rab27a (m-monoclonal; Santa Cruz; 1:100), Calnexin (r-polyclonal; Abcam; 1:1000), HSP60 (r-polyclonal; Abcam; 1:20000), VE-Cadherin (m-monoclonal; Abcam; 1:1000), Lactadherin (m-monoclonal; Santa Cruz; 1:1000) and HSP90 (m-monoclonal; Abcam; 1:1000). Bound antibodies were then detected with peroxidase-labeled anti-mouse (Calbiochem 1:5000) or anti-rabbit (Calbiochem 1:3000) IgG. Blots were revealed with the EZ-ECL system (Biological Industries) on a C-DiGit Blot Scanner (LI-COR Biosciences)^34,36^.

### Internalization assay

Exosomes (corresponding to 20 µg of protein) were suspended in 100 µl PBS and mixed with 100 µl PKH67 fluorescent dye (Sigma-Aldrich). The mix was diluted in 5 ml PBS and the exosomes were pelleted by ultracentrifugation on a 25% sucrose cushion at 100.000 × *g* for 70 min at 4 °C. The exosome pellet was washed in PBS. Finally, the pellet containing PKH67-labeled exosomes was suspended in 100 µl PBS and exosomes were quantified by NTA. Exosome internalization was determined by confocal microscopy and flow cytometry. For confocal microscopy, 1 × 10^3^ MDA-MB-231 cells were seeded per well in an 8-well lab-tek chamber slide (Nalge Nunc). The next day, 100 labeled exosomes per cell were added and incubated for 3 h at 37 °C. Slides were then stained with 2 µM CellTracker™ Red CMTPX (Life Technologies), stained with 4′,6′-diaminido-2-phenylindole (DAPI; Bio Rad). Images were taken with a 100x objective of numerical aperture 1.4 on a FV1200 Laser Scanning Microscope (Olympus). For flow cytometry, 1.5 × 10^6^ cells were seeded per well in 6-well plates (Nunc) and, on the next day, 100 labeled exosomes were added per cell. After 3 h, cells were detached by trypsinization and subjected to flow cytometry on a FACS Canto II cell sorter (BD Biosciences). Data was analyzed with the FlowJo Software v-7.6.1 (Tree star Inc, Stanford).

### Proliferation assay

Cell proliferation was determined using the MTT Cell Proliferation Assay (ATCC), according to manufacturer’s directions.

### Matrigel invasion assay

MDA-MB-231 cells were serum-starved for 24 h and then seeded over Matrigel-covered inserts (Matrigel Invasion Chamber 8.0 µm; BD Biosciences) at 1.5 × 10^5^ cells/insert. Ten µg (corresponding to protein content) of exosomes (Exo-WT, Exo-C and Exo-1537S) were added and incubation proceeded for 48 h. Inserts were then fixed in 4% PFA, stained with DAPI, washed and observed under an Olympus BX53 fluorescence microscope. At least 10 fields were evaluated per insert^36^.

### Spreading assay

MDA-MB-231 cells in suspension (1 × 10^5^) were incubated in 15 ml centrifuge tubes (Nunc) for 6 h in the presence of 10 µg total exosomes (Exo-WT, Exo-C and Exo-1537S). Afterwards, cells were allowed to attach and spread for 60 min onto coverslips coated with 2 mg/ml fibronectin. Coverslips were fixed and stained with phalloidin-Rhodamine and DAPI. At least 10 images per condition were analyzed under an Olympus BX53 fluorescent microscope. Cell spreading was evaluated on the basis of phalloidin staining, using the ImageJ software. Upon threshold adjustment, the number of pixels per image was measured and normalized to the total cell number. Cell spreading at 60 min was used as an internal standard, in order to perform the pixel-to-mm^2^ conversion, based on the scale of the image (in µm), the final quantification represents the increase in number per area.

### Colony formation assay

Anchorage-independent cell growth was determined by colony formation in soft agar as previously described^36^. Briefly, MDA-MB-231 cells were treated with 10 µg of exosomes or left untreated for 24 h. Then, cells were seeded at 2 × 10^3^ live (Tb-negative) cells/well in 12-well plates in soft agar. Formation of colonies >100 µm in diameter was scored 3 weeks after seeding under a phase-contrast microscope at 10×^36^.

### Animal studies

Animal studies were conducted in accordance with appropriate guidelines of Ethical Committee of Fundación Ciencia & Vida. Immune-compromised NOD/SCID mice were obtained from Jackson Laboratories (Bar Harbor, ME) and maintained in the animal facility of the Fundación Ciencia & Vida under specific pathogen-free conditions (Tecniplast SpA, Buguggiate, Italy), in a temperature-controlled environment with 12/12 h light/dark schedule and with sterile food and water *ad libitum*.

### Peritoneal carcinomatosis assay

Four groups of 7 NOD/SCID mice were inoculated intraperitoneally (ip) with 2.5 × 10^6^ MDA-MB-231 cells in 250 µl saline, either alone (saline group) or together with Exo-WT, Exo-C or Exo-1537S exosomes (10 µg per mouse). An additional group of 6 NOD/SCID mice was left inoculated. Animals were euthanized on day 21 post-cell injection. Mesenterial tissue and retroperitoneal tumor mass were excised and fixed in 4% *p*-formaldehide. Malignant ascites was collected and total cell number was determined by Tb exclusion assay. The final evaluation *in vivo* was performed in a double-blinded fashion.

### Proteomic analysis of exosomes

Exosomal preparations (Exo-WT, Exo-C and Exo-1537S), equivalent to 35 µg total protein, were diluted in RIPA buffer (Thermo Scientific) and sonicated in a water bath for 10 min at RT. Samples were resolved on a 10% Bis-Tris Gel (Invitrogen) and visualized using Simply Blue Safe stain (Invitrogen). Subsequently, each lane was divided into 3 fractions and each fraction was cut into approximately 1-mm gel slices. Next, gel slices were destained with 50 mM NH_4_HCO_3_ and 50 mM acetonitrile (ACN). Samples were then reduced in 10 mM DTT diluted in 100 mM NH_4_HCO_3_ and alkylated with 50 mM iodoacetic acid diluted in 100 mM NH_4_HCO_3_. Afterwards, samples were digested with Trypsin Gold (Promega) for 16 h at 37 °C. Peptides were extracted from the gel with 0.1% formic acid and dried. Next, 0.1% trifluoroacetic acid was used to reconstitute samples, which were eluted in different proportions of TFA/ACN using a C18 solid phase extraction 96 well-plate (Shimadzu). Vacuum centrifuge-dried samples were resuspended in 0.1% formic acid and stored in screw thread glass autosampler vials (Thermo Fisher). Peptides were analysed by liquid chromatography (LC)/mass spectrometry (MS) (LC-MS/MS) on a TripleTOF5600 mass spectrometer (ABSciex) equipped with an Eksigent 1D + NanoLC system with an cHiPLC system. Once injected into de column, the resulting peptide mixture was separated using a linear gradient of Buffer A containing 0.1% formic acid/water and Buffer B which contained acetonitrile/0.1% formic acid. MS/MS spectra were collected using the Information Dependent Acquisition (IDA) with a survey scan (m/z 350–1500) along with 25 data-dependent product ion scans of the 25 most intense precursor ions. All mass spectra were analyzed using Protein PilotTM software with the Paragon method as a search engine against the Uniprot-database (setting human as default species). False discovery rate (FDR) was estimated using a reversed sequence database. Data was subjected to ontology and pathway analysis using the protein analysis through evolutionary relationships tool (PANTHER) and gene ontology algorithms and classified based on pathway, biological process and molecular function categories.

### Statistical analysis

The Kruskal–Wallis test was used, followed by Dunn’s multiple comparison post-test. For invasion and *in vivo* experiments, results were compared using unpaired *t*-tests and corroborated with Tukey by multiple comparison post-test. Quantitative Western blot analysis was analyzed using unpaired *t*-test and one-way ANOVA.

## Supplementary information

Supplementary Figures S1, S2 and S3.

## References

[1] Siegel RL, Miller KD, Jemal A (2019). Cancer statistics, 2019. CA Cancer J. Clin..

[2] Terrell-Hall TB, Nounou MI, El-Amrawy F, Griffith JIG, Lockman PR (2017). Trastuzumab distribution in an *in-vivo* and *in-vitro* model of brain metastases of breast cancer. Oncotarget.

[3] Grubb W, Young R, Efird J, Jindal C, Biswas T (2017). Local therapy for triple-negative breast cancer: a comprehensive review. Future Oncol..

[4] Sotgia F, Fiorillo M, Lisanti MP (2017). Mitochondrial markers predict recurrence, metastasis and tamoxifen-resistance in breast cancer patients: Early detection of treatment failure with companion diagnostics. Oncotarget.

[5] Weigelt B, Peterse JL, van ‘t Veer LJ (2005). Breast cancer metastasis: markers and models. Nat. Rev. Cancer.

[6] Polyak K (2014). Heterogeneity in breast cancer. J. Clin. Invest..

[7] Banin Hirata BK (2014). Molecular markers for breast cancer: prediction on tumor behavior. Dis. Markers.

[8] Subik K (2010). The Expression Patterns of ER, PR, HER2, CK5/6, EGFR, Ki-67 and AR by Immunohistochemical Analysis in Breast Cancer Cell Lines. Breast Cancer (Auckl.).

[9] Sueta A (2017). Differential expression of exosomal miRNAs between breast cancer patients with and without recurrence. Oncotarget.

[10] Cleary AS, Leonard TL, Gestl SA, Gunther EJ (2014). Tumour cell heterogeneity maintained by cooperating subclones in Wnt-driven mammary cancers. Nature.

[11] Junttila MR, de Sauvage FJ (2013). Influence of tumour micro-environment heterogeneity on therapeutic response. Nature.

[12] Jia, Y. *et al*. Exosome: emerging biomarker in breast cancer. *Oncotarget* (2017). 10.18632/oncotarget.16684 PMC5522217 28402944

[13] Joyce DP, Kerin MJ, Dwyer RM (2016). Exosome-encapsulated microRNAs as circulating biomarkers for breast cancer. Int. J. Cancer.

[14] Eichelser C (2014). Increased serum levels of circulating exosomal microRNA-373 in receptor-negative breast cancer patients. Oncotarget.

[15] Camussi G (2011). Exosome/microvesicle-mediated epigenetic reprogramming of cells. Am. J. Cancer Res..

[16] Valadi H (2007). Exosome-mediated transfer of mRNAs and microRNAs is a novel mechanism of genetic exchange between cells. Nat. Cell Biol..

[17] Andaloussi S, Mager I, Breakefield XO, Wood MJ (2013). Extracellular vesicles: biology and emerging therapeutic opportunities. Nat. Rev. Drug. Discov..

[18] Lee Y, El Andaloussi S, Wood MJ (2012). Exosomes and microvesicles: extracellular vesicles for genetic information transfer and gene therapy. Hum. Mol. Genet..

[19] Palazzolo G (2012). Proteomic analysis of exosome-like vesicles derived from breast cancer cells. Anticancer. Res..

[20] Rana S, Malinowska K, Zoller M (2013). Exosomal tumor microRNA modulates premetastatic organ cells. Neoplasia.

[21] Rak J, Guha A (2012). Extracellular vesicles–vehicles that spread cancer genes. Bioessays.

[22] Ono M (2014). Exosomes from bone marrow mesenchymal stem cells contain a microRNA that promotes dormancy in metastatic breast cancer cells. Sci. Signal..

[23] Gorczynski RM, Erin N, Zhu F (2016). Serum-derived exosomes from mice with highly metastatic breast cancer transfer increased metastatic capacity to a poorly metastatic tumor. Cancer Med..

[24] Hong BS (2009). Colorectal cancer cell-derived microvesicles are enriched in cell cycle-related mRNAs that promote proliferation of endothelial cells. BMC Genomics.

[25] Peinado H (2012). Melanoma exosomes educate bone marrow progenitor cells toward a pro-metastatic phenotype through MET. Nat. Med..

[26] Galindo-Hernandez O, Serna-Marquez N, Castillo-Sanchez R, Salazar EP (2014). Extracellular vesicles from MDA-MB-231 breast cancer cells stimulated with linoleic acid promote an EMT-like process in MCF10A cells. Prostaglandins Leukot. Essent. Fat. Acids.

[27] Qin, W. *et al*. Exosomes in Human Breast Milk Promote EMT. *Clin Cancer Res* (2016). 10.1158/1078-0432.CCR-16-0135 27060153

[28] Lobb RJ, Lima LG, Moller A (2017). Exosomes: Key mediators of metastasis and pre-metastatic niche formation. Semin. Cell Dev. Biol..

[29] Liu Y, Cao X (2016). Organotropic metastasis: role of tumor exosomes. Cell Res..

[30] Feng Q (2017). A class of extracellular vesicles from breast cancer cells activates VEGF receptors and tumour angiogenesis. Nat. Commun..

[31] Rong L, Li R, Li S, Luo R (2016). Immunosuppression of breast cancer cells mediated by transforming growth factor-beta in exosomes from cancer cells. Oncol. Lett..

[32] Cho JA, Park H, Lim EH, Lee KW (2012). Exosomes from breast cancer cells can convert adipose tissue-derived mesenchymal stem cells into myofibroblast-like cells. Int. J. Oncol..

[33] Luga V (2012). Exosomes mediate stromal mobilization of autocrine Wnt-PCP signaling in breast cancer cell migration. Cell.

[34] Villegas J (2007). Expression of a novel non-coding mitochondrial RNA in human proliferating cells. Nucleic Acids Res..

[35] Vidaurre S (2014). Down-regulation of the antisense mitochondrial non-coding RNAs (ncRNAs) is a unique vulnerability of cancer cells and a potential target for cancer therapy. J. Biol. Chem..

[36] Lobos-Gonzalez L (2016). Targeting antisense mitochondrial ncRNAs inhibits murine melanoma tumor growth and metastasis through reduction in survival and invasion factors. Oncotarget.

[37] Varas-Godoy M (2018). *In vivo* knockdown of antisense non-coding mitochondrial RNAs by a lentiviral-encoded shRNA inhibits melanoma tumor growth and lung colonization. Pigment. Cell Melanoma Res..

[38] Fitzpatrick C (2019). Mitochondrial ncRNA targeting induces cell cycle arrest and tumor growth inhibition of MDA-MB-231 breast cancer cells through reduction of key cell cycle progression factors. Cell Death Dis..

[39] Morrison Joly M (2017). Two distinct mTORC2-dependent pathways converge on Rac1 to drive breast cancer metastasis. Breast Cancer Res..

[40] Yang, S.J. *et al*. Predictive role of GSTP1-containing exosomes in chemotherapy-resistant breast cancer. *Gene* (2017). 10.1016/j.gene.2017.04.031 28438694

[41] Borgna V (2017). Mitochondrial ASncmtRNA-1 and ASncmtRNA-2 as potent targets to inhibit tumor growth and metastasis in the RenCa murine renal adenocarcinoma model. Oncotarget.

[42] Wedin R, Skoog L, Bauer HC (2004). Proliferation rate, hormone receptor status and p53 expression in skeletal metastasis of breast carcinoma. Acta Oncol..

[43] Oshima K, Aoki N, Kato T, Kitajima K, Matsuda T (2002). Secretion of a peripheral membrane protein, MFG-E8, as complex with membrane vesicles. Eur. J. Biochem..

[44] Fidler IJ, Kripke ML (2015). The challenge of targeting metastasis. Cancer Metastasis Rev..

[45] Harris DA (2015). Exosomes released from breast cancer carcinomas stimulate cell movement. PLoS One.

[46] Al-Nedawi K, Meehan B, Kerbel RS, Allison AC, Rak J (2009). Endothelial expression of autocrine VEGF upon the uptake of tumor-derived microvesicles containing oncogenic EGFR. Proc. Natl Acad. Sci. USA.

[47] Hoshino A (2015). Tumour exosome integrins determine organotropic metastasis. Nature.

[48] Sung BH, Ketova T, Hoshino D, Zijlstra A, Weaver AM (2015). Directional cell movement through tissues is controlled by exosome secretion. Nat. Commun..

[49] Fei F (2017). Role of metastasis-induced protein S100A4 in human non-tumor pathophysiologies. Cell Biosci..

[50] Fei F, Qu J, Zhang M, Li Y, Zhang S (2017). S100A4 in cancer progression and metastasis: A systematic review. Oncotarget.

[51] Bresnick AR, Weber DJ, Zimmer DB (2015). S100 proteins in cancer. Nat. Rev. Cancer.

[52] Taylor MR, Couto JR, Scallan CD, Ceriani RL, Peterson JA (1997). Lactadherin (formerly BA46), a membrane-associated glycoprotein expressed in human milk and breast carcinomas, promotes Arg-Gly-Asp (RGD)-dependent cell adhesion. DNA Cell Biol..

[53] Neutzner M (2007). MFG-E8/lactadherin promotes tumor growth in an angiogenesis-dependent transgenic mouse model of multistage carcinogenesis. Cancer Res..

[54] Carrascosa C (2012). MFG-E8/lactadherin regulates cyclins D1/D3 expression and enhances the tumorigenic potential of mammary epithelial cells. Oncogene.

[55] Sugano G (2011). Milk fat globule–epidermal growth factor–factor VIII (MFGE8)/lactadherin promotes bladder tumor development. Oncogene.

[56] Tibaldi L (2013). New blocking antibodies impede adhesion, migration and survival of ovarian cancer cells, highlighting MFGE8 as a potential therapeutic target of human ovarian carcinoma. PLoS One.

[57] Fry SA, Sinclair J, Timms JF, Leathem AJ, Dwek MV (2013). A targeted glycoproteomic approach identifies cadherin-5 as a novel biomarker of metastatic breast cancer. Cancer Lett..

[58] Higuchi K (2017). Cadherin 5 expression correlates with poor survival in human gastric cancer. J. Clin. Pathol..

[59] Inokuchi M (2017). Cadherin 5 Is a Significant Risk Factor for Hematogenous Recurrence and a Prognostic Factor in Locally Advanced Gastric Cancer. Anticancer. Res..

[60] Bartolome RA (2017). VE-cadherin RGD motifs promote metastasis and constitute a potential therapeutic target in melanoma and breast cancers. Oncotarget.

[61] Rochefort P (2017). Soluble VE-cadherin in metastatic breast cancer: an independent prognostic factor for both progression-free survival and overall survival. Br. J. Cancer.

[62] Ding J (2018). Exosome-mediated miR-222 transferring: An insight into NF-kappaB-mediated breast cancer metastasis. Exp. Cell Res..

[63] Wen SW (2016). The Biodistribution and Immune Suppressive Effects of Breast Cancer-Derived Exosomes. Cancer Res..

