# Supplementary material for: Exosomes released upon mitochondrial ASncmtRNA knockdown reduce tumorigenic properties of malignant breast cancer cells
Source: Sci Rep. 2020 Jan 15;10:343. doi: 10.1038/s41598-019-57018-1 (PMC6962334; doi:10.1038/s41598-019-57018-1)
Supplement: Supplementary file 1 — Supplementary Figures S1, S2 and S3. [file 41598_2019_57018_MOESM1_ESM.pdf]

Exosomes released upon mitochondrial ASncmtRNA knockdown reduce tumorigenic properties of malignant breast cancer cells.

Lorena Lobos-González, Rocío Bustos, América Campos, Valeria Silva, Verónica Silva, Emanuel Jeldes, Carlos Salomon, Manuel Varas-Godoy, Albano Cáceres-Verschae, Eduardo Duran, Tamara Vera, Fernando Ezquer, Marcelo Ezquer, Verónica A. Burzio, Jaime Villegas.

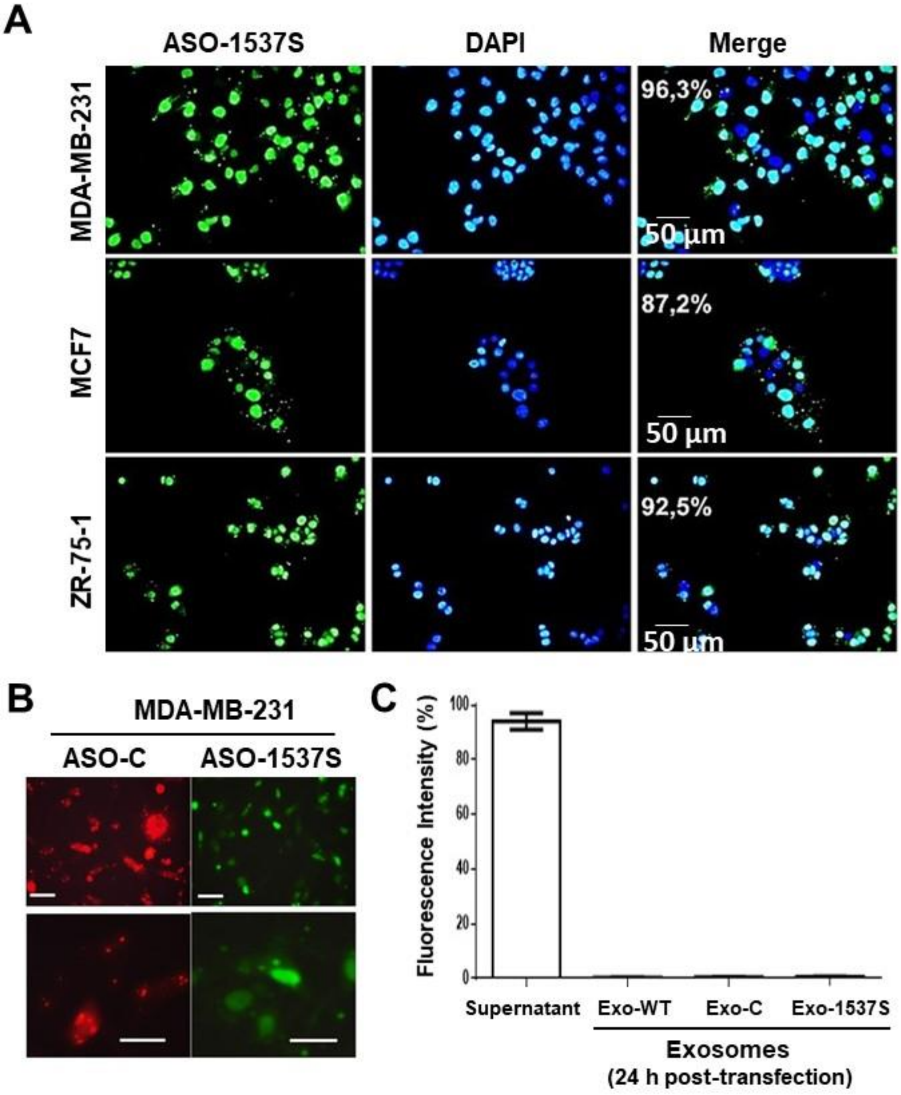

Supplementary Figure S1: Antisense Oligonucleotides are not incorporated into exosomes. A) MDA-MB-231, MCF7 and ZR-75 breast cancer cells were transfected for 24 h with Alexa 488-ASO-C and % transfected cells was determined by fluorescence microscopy. Representative images are shown (magnification 40x). **B)** MDA-MB-231 cells were transfected for 24 h with Alexa 488-ASO-C and Cy3-ASO-1537S. Transfection efficiency was monitored by fluorescence microscopy. The efficiency of transfection ranged between 87 ± 3 % and 96 ± 4 %. Bars = 50 µm. **C)** At 24 h post-transfection, supernatants in all conditions were recovered and exosomes were purified. Fluorescence intensity was measured in exosome purifications and in the supernatant using a Horiba Fluorimeter (Japan). Upper panels 40X magnification, lower panels correspond to digital magnification. Bars = 50 µm.

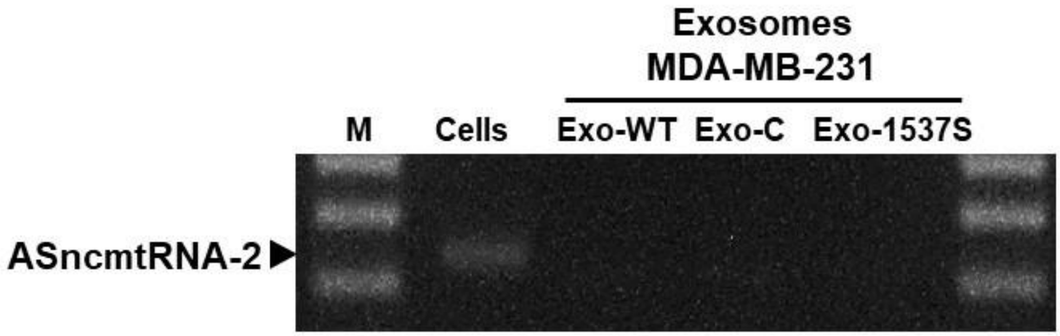

Supplementary Figure S2. Antisense noncoding mitochondrial RNA-2 is not incorporated into exosomes. Total RNA was purified from: Exo-WT, Exo-C and Exo-1537S exosomes using TRIzol and ASncmtRNA-2 was amplified by RT-PCR.

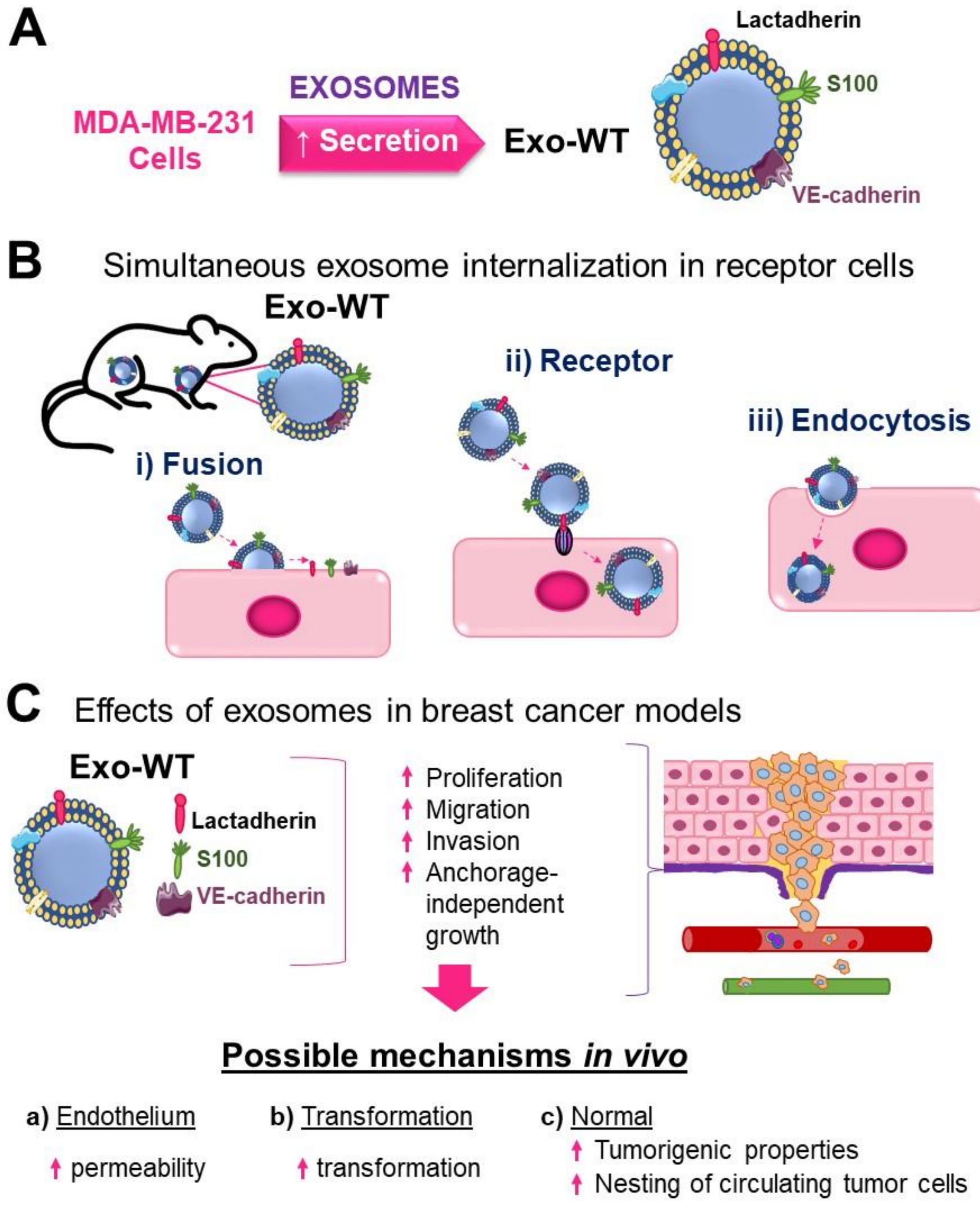

Supplementary Figure S3. Hypothetical mechanism of the action of exosomes in the MDA-MB-231 breast cancer model. A) MDA-MB-231 cells secrete exosomes that contain tumor promotor proteins, specifically Lactadherin, VE-cadherin and S100. B) Circulating exosomes are incorporated into receptor cells by membrane fusion, receptor-mediated internalization and endocytosis. C) Lactadherin, VE-cadherin and S100 modify the properties of receptor cells such as anchorage-independent growth, increase in proliferative index, migration and invasion. The increase in metastasis could be explained by three different additional acquired capacities; increase in cellular and endothelial permeability, cellular transformation, promotion of tumorigenic properties and generation of new metastatic niches.
